# BO-CNN-BiLSTM deep learning model integrating multisource remote sensing data for improving winter wheat yield estimation

**DOI:** 10.3389/fpls.2024.1500499

**Published:** 2024-12-20

**Authors:** Lei Zhang, Changchun Li, Xifang Wu, Hengmao Xiang, Yinghua Jiao, Huabin Chai

**Affiliations:** ^1^ School of Surveying and Land Information Engineering, Henan Polytechnic University, Jiaozuo, China; ^2^ Shandong Provincial Land Survey and Planning Institute, Jinan, Shandong, China

**Keywords:** bidirectional long short-term memory (BiLSTM), 1D convolutional neural network (1D CNN), Bayesian optimization (BO), solar-induced chlorophyll fluorescence (SIF), yield estimation

## Abstract

**Introduction:**

In the context of climate variability, rapid and accurate estimation of winter wheat yield is essential for agricultural policymaking and food security. With advancements in remote sensing technology and deep learning, methods utilizing remotely sensed data are increasingly being employed for large-scale crop growth monitoring and yield estimation.

**Methods:**

Solar-induced chlorophyll fluorescence (SIF) is a new remote sensing metric that is closely linked to crop photosynthesis and has been applied to crop growth and drought monitoring. However, its effectiveness for yield estimation under various data fusion conditions has not been thoroughly explored. This study developed a deep learning model named BO-CNN-BiLSTM (BCBL), combining the feature extraction capabilities of a convolutional neural network (1DCNN) with the time-series memory advantages of a bidirectional long short-term memory network (BiLSTM). The Bayesian Optimization (BOM) method was employed to determine the optimal hyperparameters for model parameter optimization. Traditional remote sensing variables (TS), such as the Enhanced Vegetation Index (EVI) and Leaf Area Index (LAI), were fused with the SIF and climate data to estimate the winter wheat yields in Henan Province, exploring the SIF’s estimation capabilities using various datasets.

**Results and Discussion:**

The results demonstrated that the BCBL model, integrating TS, climate, and SIF data, outperformed other models (e.g., LSTM, Transformer, RF, and XGBoost) in the estimation accuracy, with R^²^=0.81, RMSE=616.99 kg/ha, and MRE=7.14%. Stepwise sensitivity analysis revealed that the BCBL model reliably identified the critical stage of winter wheat yield formation (early March to early May) and achieved high yield estimation accuracy approximately 25 d before harvest. Furthermore, the BCBL model exhibited strong stability and generalization across different climatic conditions.

**Conclusion:**

Thus, the BCBL model combined with SIF data can offer reliable winter wheat yield estimates, hold significant potential for application, and provide valuable insights for agricultural policymaking and field management.

## 1 Introduction

Wheat is one of the most important staple crops worldwide, not only serving as a primary food source for humans but also playing a crucial role as livestock feed and an industrial raw material, making it essential for global food security (Langridge et al., 2022). Winter wheat holds a central position in agricultural production and food reserves in many countries. However, abnormal climate conditions in recent years, including reduced precipitation and abnormal temperature increases, have led to frequent droughts, posing a serious threat to wheat yields (Farooq et al., 2023; Rezaei et al., 2023). Drought has become a primary factor limiting winter wheat yield, directly impacting agricultural income and the stability of the food supply chain (Yu et al., 2018). In this context, accurate crop yield estimation is not only significant for national food security and policy making but also serves as a critical decision-making tool in the futures market. Accurate yield predictions can help governments and agricultural practitioners formulate proactive responses to mitigate risks of price volatility in the food market (Feng et al., 2020). Therefore, accurately estimating wheat yields under variable climatic conditions has become a significant challenge.

Traditional methods for field and regional yield estimation rely heavily on manual field surveys, which are labor-intensive and limited in scale. Alternatively, crop growth models such as DSSAT, APSIM, WOFOST, and PCSE, which depend on extensive crop growth data (Huang et al., 2019; Jin et al., 2018; Martre et al., 2015), can accurately simulate crop growth and interactions between climate and soil factors at the field scale. These models provide valuable guidance for production management and risk assessments. However, their application at larger scales is constrained by the spatial heterogeneity of soil properties, climate factors, crop parameters, and field management strategies, making large-scale deployment challenging (Li et al., 2019). Recent advancements in remote sensing technology have enabled large-scale crop growth monitoring, rendering it the preferred method for regional-scale yield estimation (Cao et al., 2021; Huang et al., 2015; Li et al., 2020). Satellites capture various spectral bands, including visible, near-infrared, thermal infrared, and microwave bands, to assess crop growth conditions and estimate yields (Guan et al., 2017). Combining these spectral bands allows for the calculation of vegetation indices, such as the Normalized Difference Vegetation Index (NDVI) and Enhanced Vegetation Index (EVI), as well as the indicators related to crop yield and photosynthesis, including the Fraction of Photosynthetically Active Radiation (FPAR) and Leaf Area Index (LAI) (Huang et al., 2016; Xie et al., 2017). LAI, which is closely related to crop growth and yield accumulation, represents crop biomass and photosynthesis, whereas EVI, which minimizes atmospheric and soil noise, is widely used to monitor vegetation growth and coverage.

Owing to the complex nonlinear relationship between climate change and crop yield formation, linear models often fail to capture these dynamic processes. Machine learning algorithms, such as Random Forest (RF) and Extreme Gradient Boosting (XGBoost), are effective in handling nonlinear data and have demonstrated stability and accuracy in land cover classification and yield estimation (Chlingaryan et al., 2018; Fei et al., 2023). Deep learning, which leverages multilayer structures for feature extraction, can model intricate nonlinear relationships and has been applied in areas such as image recognition and natural language processing (Bhatt et al., 2021; Noda et al., 2015; Yang et al., 2018). Remote sensing data-driven deep-learning methods have markedly improved the monitoring of crop growth and yield estimation, typically utilizing time-series data from the entire growing season as the model input. Recurrent Neural Networks (RNNs) are well suited for time-series data particularly the Long Short-Term Memory (LSTM) model, effectively address vanishing and exploding gradient issues (Tian et al., 2021). Cao et al. (2021) utilized an LSTM model incorporating climate, satellite, soil, and spatial information to predict county-level winter wheat yields in China’s major wheat-producing regions (Cao et al., 2021). Wang et al. (2020) developed a dual-branch CNN-LSTM model that integrated climate, remote sensing, and soil data for estimating wheat yields in the same regions, demonstrating superior performance compared to standalone CNN or LSTM models and highlighting the benefits of hybrid approaches (Wang et al., 2020). Hybrid models generally provide more accurate results for complex data than single models. Wang et al. (2023) introduced a multilayer CNN-GRU yield estimation framework, which effectively estimated the spatial and temporal distribution of yields on the Guanzhong Plain. Given the complexity of deep learning model parameters, relying only on past experience for setting these parameters can impact accuracy (Wang et al., 2023). Consequently, further exploration of optimization algorithms in deep learning models is essential to enhance their accuracy.

Compared with traditional vegetation indices such as NDVI, EVI, and LAI, Solar-Induced Chlorophyll Fluorescence (SIF) is more sensitive to photosynthesis (Dechant et al., 2022, 2020) and demonstrates higher sensitivity under drought conditions (Wang et al., 2022). Luo et al. (2024) used the high-resolution SIF combined with traditional indices (LAI, EVI) to predict yields in the US Corn Belt, indicating that the combination of SIF with traditional indices yields better performance than using either index alone (Luo et al., 2024). Other studies have highlighted the potential of SIF for monitoring drought stress and estimating crop yields (He et al., 2019). Furthermore, the model performance varies with different combinations of remote sensing variables, indicating the need for further exploration of the predictive performance of SIF in various combinations.

The application of multimodal remote sensing data fusion methods in crop growth monitoring and yield estimation has developed rapidly, especially in predicting crop health and yield under complex climate conditions. To address the effective fusion of data from various remote sensing sources, several advanced deep learning methods have recently been proposed, offering new insights for agricultural remote sensing applications. First, the Vision Transformer (ViT) has demonstrated superior feature-capturing capabilities in multimodal data fusion due to its self-attention mechanism. Recent studies, such as the Morphological Transformer (morphFormer), have enhanced spectral-spatial representation of images by incorporating spectral and spatial convolution operations, showcasing stronger spectral and spatial information extraction capabilities (Roy et al., 2023). This method’s success highlights that combining attention mechanisms with convolution operations can significantly improve the application of remote sensing data in complex tasks. Moreover, multimodal frameworks like the Extended Vision Transformer (ExViT) use parallel branch structures and cross-modal attention mechanisms to achieve feature fusion of multisource data, effectively handling misaligned data in remote sensing (Yao et al., 2023). This approach has broad potential in crop monitoring, particularly when analyzing multisource information in crop growth (such as SIF, EVI, LAI, and meteorological data), as it improves classification and prediction performance by integrating modal features. Additionally, unsupervised methods based on low-rank diffusion models have shown advantages in remote sensing data sharpening and spatial resolution enhancement (Rui et al., 2024). By combining low-rank tensor decomposition with Bayesian optimization techniques, this approach overcomes traditional deep learning’s dependency on large-scale labeled data, achieving high-accuracy feature extraction even in data-scarce scenarios and supporting the broader adoption of multimodal data in agricultural applications. These innovative multimodal data fusion techniques, employing convolutional neural networks, attention mechanisms, Bayesian optimization, and low-rank decomposition, enhance the processing of complex spatiotemporal information from multisource remote sensing data, providing new tools and directions for tasks like winter wheat yield estimation. The advancement of these technologies lays a solid foundation for improving crop yield estimation accuracy and addressing the complex challenges posed by climate change.

In summary, this study developed a BO-CNN-BiLSTM (BCBL) deep learning model that integrated SIF, EVI, LAI, and climate data, combining the CNN’s spatial feature extraction with the BiLSTM’s sequence data capture to predict the winter wheat yield in Henan Province from 2011 to 2020. The performance of the BCBL model was compared with those of the LSTM, RF, and XGBoost models. The primary research objectives were (1) to evaluate the accuracy of the BCBL model for county-level yield estimation and compare it with other models, (2) to explore the predictive performance of SIF under various datasets and its impact on early yield estimation accuracy, and (3) to analyze the spatial-temporal distribution of estimated yields from 2015 to 2020 using the BCBL model.

## 2 Materials and methods

### 2.1 Study area

**Figure 1:**
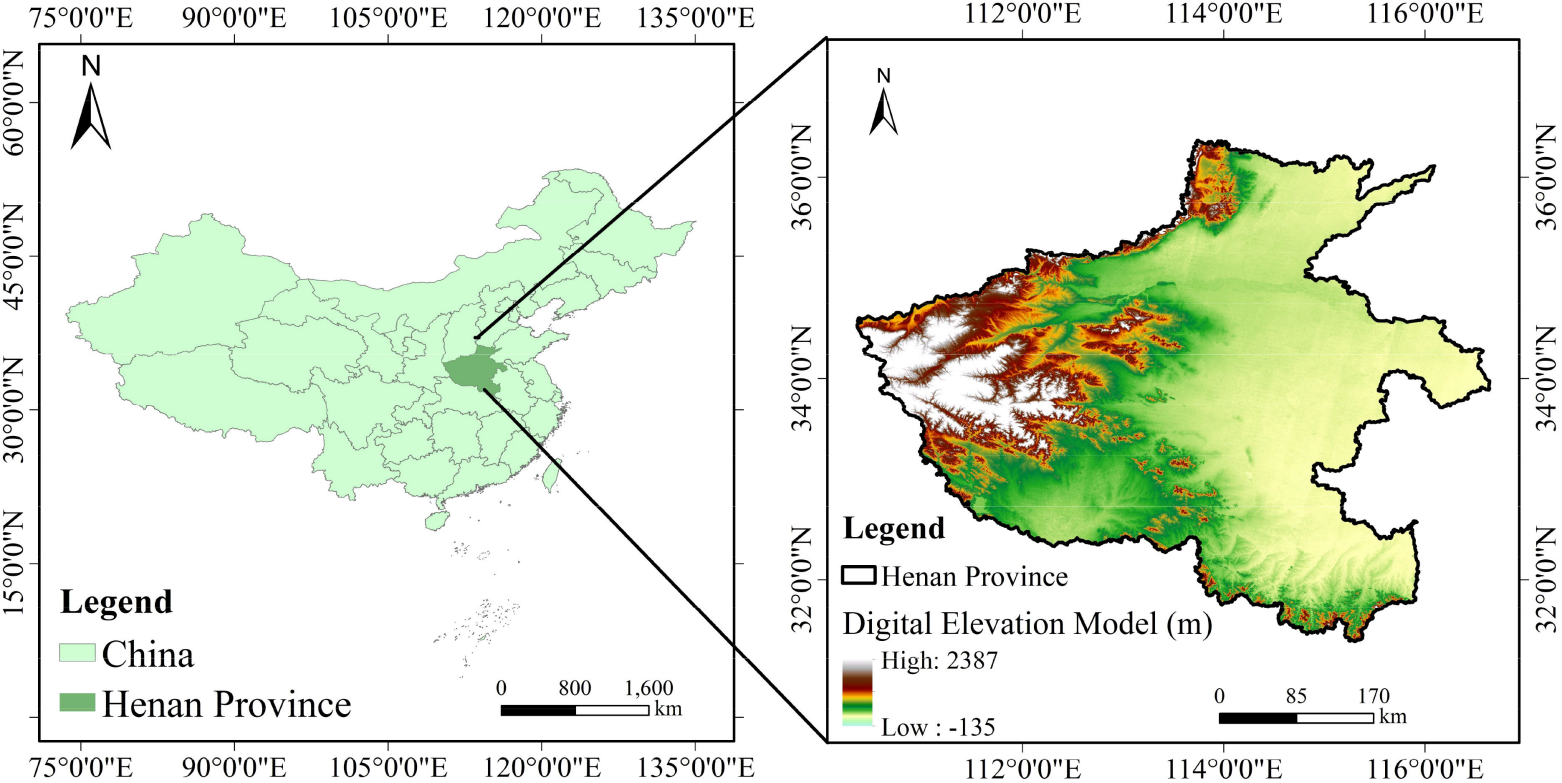
Location and topography of the study area.

The study area is located in Henan Province, China, with geographical coordinates ranging from 110°21′E to 116°39′E and 31°23′N to 36°22′N (**Figure 1**). The terrain slopes from high elevation in the west to low elevation in the east. Most of the area is within a warm temperate zone, whereas the southern part extends into the subtropical zone, characterized by a continental monsoon climate transitioning from the northern subtropical to the warm temperate zone. The region experienced four distinct seasons with simultaneous rainfall and heat and had a complex and diverse climate. The fertile soil makes the area highly suitable for agriculture. Henan Province is a major grain-producing region in China, contributing more than 20% of the national wheat yield and consistently ranking first. Winter wheat was sown in early October and harvested in early June of the following year. The entire growing period of winter wheat was selected for modeling and analysis in this study. Among the major natural disasters in Henan Province, droughts and floods have the most serious impact on agriculture, often causing significant losses to winter wheat crops.

### 2.2 Dataset and preprocessing

#### 2.2.1 SIF

Solar-Induced Chlorophyll Fluorescence (SIF) has emerged as a promising remote sensing index in recent years owing to its close coupling with photosynthesis, demonstrating its superior performance in agricultural monitoring and environmental assessment (Zhang et al., 2018). Compared with traditional vegetation indices such as the Normalized Difference Vegetation Index (NDVI) and Enhanced Vegetation Index (EVI), SIF offers more direct information about photosynthesis, making it advantageous for crop yield estimation and drought monitoring (Sun et al., 2015; Mohammed et al., 2019; Qiu et al., 2022). This study utilized a global SIF dataset from the National Tibetan Plateau Data Center (https://data.tpdc.ac.cn/) provided by Yao (Yao, 2021), which covers SIF observations on a global scale (4d, 0.05°×0.05°). In this study, the raw data were resampled using bilinear interpolation with a spatial resolution of 500 metres and the coordinate system was converted to WGS-84 to improve accuracy and applicability. To ensure the image time alignment problem. Images with the same or similar dates as the start of winter wheat sowing in EVI and LAI were selected, and the maximum value compositing (MVC) method was used to generate an 8-day time series image of winter wheat during the growing period, and cloud cover and atmospheric disturbances were effectively reduced by selecting the maximum SIF value. County-level wheat SIF values were then averaged to obtain the mean SIF for each region during the growth period, providing a more accurate assessment of photosynthesis and solid foundation for subsequent yield estimation and drought monitoring.

#### 2.2.2 LAI and EVI

Two other satellite data products commonly used in crop yield estimation, including the Leaf Area Index (LAI) and Enhanced Vegetation Index (EVI), were selected. Research has indicated that LAI, representing the total leaf area per unit of land area, can be closely related to crop growth and yield accumulation, making it a key indicator of vegetation coverage. An EVI optimized to minimize atmospheric and soil noise can be widely adopted for monitoring vegetation growth and coverage. However, the original MODIS LAI (MOD15A2H, 8d, 500m) and MODIS EVI (MOD13A1-16d,500m and MOD13A1-16d,500m combined as 8d, 500m) products often contain discontinuities and noise owing to cloud and precipitation interference (https://lpdaac.usgs.gov/). To address this issue, a Savitzky-Golay (Window Size=9, Polynomial Order=2) filter (Chen et al., 2004) was applied to smooth the raw data pixel-by-pixel, effectively reducing noise while preserving primary trends. Subsequently, county-level LAI and EVI values were averaged over an 8-day time series with a spatial resolution of 500m using the winter wheat planting period as the first day to improve the accuracy and reliability of the data.

#### 2.2.3 Climate data

The interaction between climate change and wheat yields is complex. The climate data used in this study were derived from ERA5(1d, 0.1°×0.1°), a fifth-generation atmospheric reanalysis dataset produced by the European Center for Medium-Range Weather Forecasts (ECMWF, https://www.ecmwf.int/). ERA5 combined the model outputs with global observations to provide a comprehensive global dataset, including the 2-meter air temperature (T2m), minimum and maximum air temperatures (Tmn and Tmx), total precipitation (Pre), the 10-meter u-component wind speed (U10m), and the 10-meter v-component wind speed (V10m). For climatic data with shorter time intervals (1 day), they were converted to 8-day temporal resolution by data aggregation (8-day aggregated averaging) based on the start dates of other datasets, and the coordinate system was converted to WGS-84 to ensure consistency with other datasets. Secondly, winter wheat meteorological data were obtained by masking method using winter wheat distribution information, and finally aggregated to county-level wheat meteorological data by averaging method. The 2-meter air temperature and temperature range were crucial for understanding crop growth, as they could affect photosynthesis and growth rates. The total precipitation affected the soil moisture and water availability for wheat, and the wind components could provide insights into evaporation rates and potential extreme climate events. Analyzing these variables is essential for understanding the impact of climate change on wheat yield. Additionally, we used the Standardized Precipitation Evapotranspiration Index (SPEI) to represent drought conditions across different time zones in the study area. The SPEI data (https://spei.csic.es/index.html) provides global long-term drought information with a spatial resolution of 0.5 degrees and a monthly temporal resolution. This dataset features multi-scale properties, offering SPEI time scales from 1 month to 48 months. We utilized the SPEIbase v2.9 version, with data spanning from January 1901 to December 2022. For our study area, we obtained monthly SPEI data from 2010 to 2020 at a spatial resolution of 0.5°×0.5°. Drought classification according to SPEI is provided in **Table 1**.

**Table 1:** SPEI classification criteria.

Value range	SPEI > -0.5	-0.5≥SPEI>-1	-1≥SPEI>-1.5	-1.5≥SPEI>-2	SPEI≤-2
Drought level	Non-drought (ND)	Moderate drought (MD1)	Middle drought (MD2)	Severe Drought (SD)	Extreme Drought (ED)

#### 2.2.4 Yield data and area

Reliable winter wheat yield data are essential for enhancing the accuracy of model yield estimates. This study used county-level winter wheat yield data from the National Bureau of Statistics of China (https://www.stats.gov.cn/), focusing on 10 consecutive years and selecting counties with yield levels above 3,000 kg/ha for analysis. After screening, 93 counties were included in each year. Statistical yearbook data indicated that the annual variation in the winter wheat planting area was less than 1%, with winter wheat being the primary crop from October to early June. The cropland data from the land use classification were applied as a mask for winter wheat, with the MODIS land classification product (MCD12Q1) providing yearly images and selecting land cover types based on the IGBP global vegetation classification scheme. These data were processed using the Google Earth Engine (GEE), and annual winter wheat planting distribution maps for each county were generated in combination with county administrative boundary vector files.

### 2.3 Methods

#### 2.3.1 Experimental technical approach

**Figure 2:**
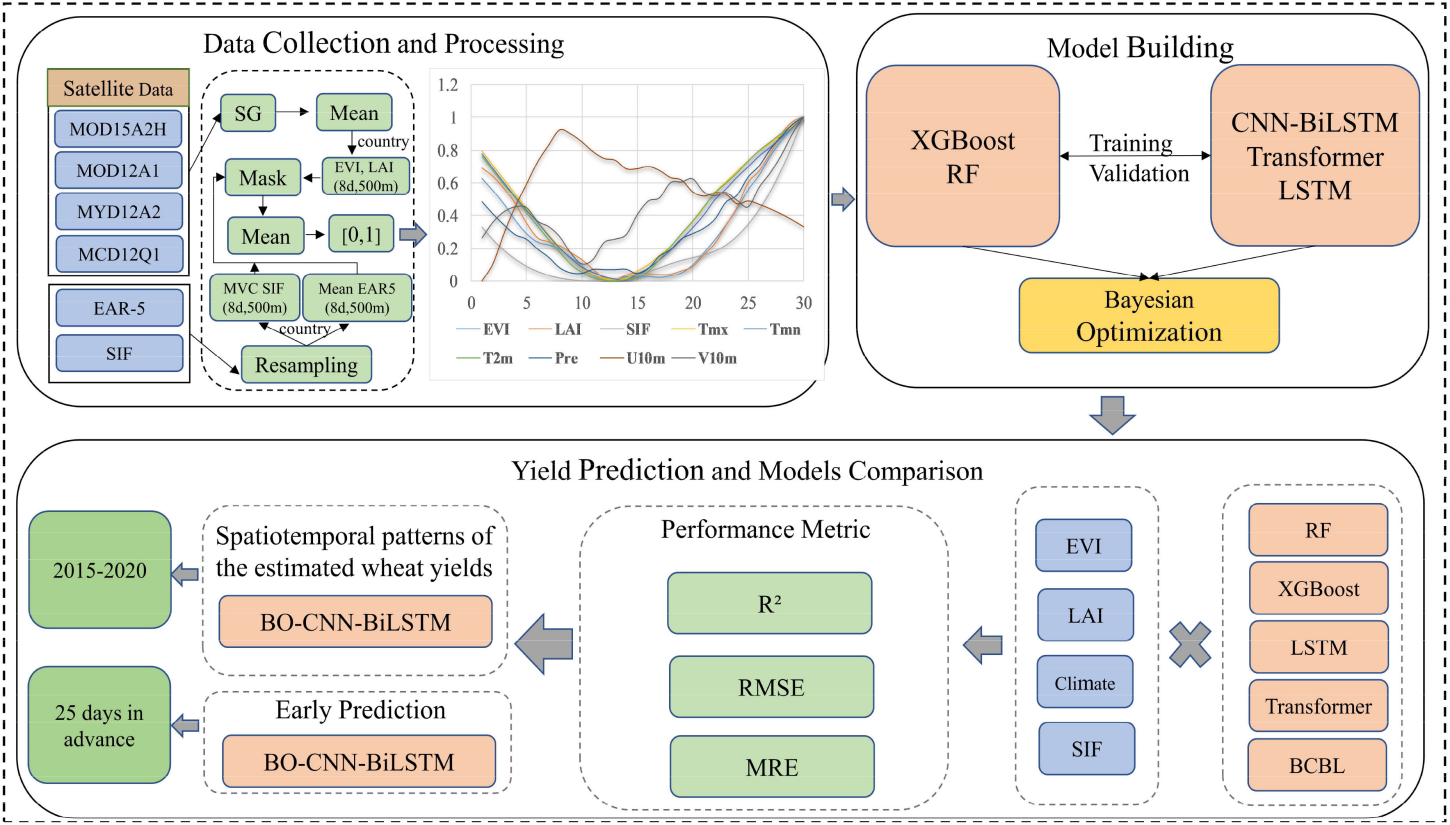
Flowchart of research techniques.

The experimental approach in this study was divided into three stages (**Figure 2**): data collection and processing, model construction comparison, and yield prediction. Initially, multisource remote sensing data (including MOD15A2H, MOD12A1, MYD12A2, and MCD12Q1), climate data (EAR-5), and the novel remote sensing index SIF for Henan Province from 2011 to 2020 were collected. During data preprocessing, all data were resampled to an 8-day interval with a 500 m resolution, and the Savitzky-Golay filter was adopted to smooth the time series data(EVI, LAI). The mean of each feature was then calculated, the target area wheat data were extracted through mask processing, and all features were normalized to a range of [0, 1] to prepare them for the model input.

In the model construction and yield prediction stage, various models, including traditional algorithms such as RF and XGBoost, as well as certain deep learning models such as BO-CNN-BiLSTM (BCBL) and baseline LSTM、Transformer, were trained using the preprocessed data. Bayesian Optimization was employed to fine-tune the hyperparameters of the models and enhance their estimation accuracy. The study then performed a spatiotemporal analysis of winter wheat yield from 2015 to 2020 and assessed the predictive ability of the models, with a focus on the accuracy of predictions made 25 d prior to harvest. The prediction accuracy of each model was compared using performance metrics, such as R², RMSE, and MRE. Additionally, the role of SIF data in enhancing the accuracy of traditional remote sensing data was analyzed, with a particular emphasis on its performance under extreme climate conditions.

#### 2.3.2 BiLSTM network

Bidirectional Long Short-Term Memory (BiLSTM) is an advanced version of the Long Short-Term Memory (LSTM) network designed to effectively handle time-series data while preserving memory. Based on LSTM’s solution to the vanishing and exploding gradient problems inherent in Recurrent Neural Networks (RNNs), BiLSTM can enhance time-series modeling by running two LSTM networks simultaneously. One network can process the data forward in time, and the other processes it backward, enabling the consideration of both past and future information.

The key components of LSTM include cell states and several gating mechanisms: forget, input, and output gates. These mechanisms can regulate the flow of information between different gates using a sigmoid function.

**Figure 3:**
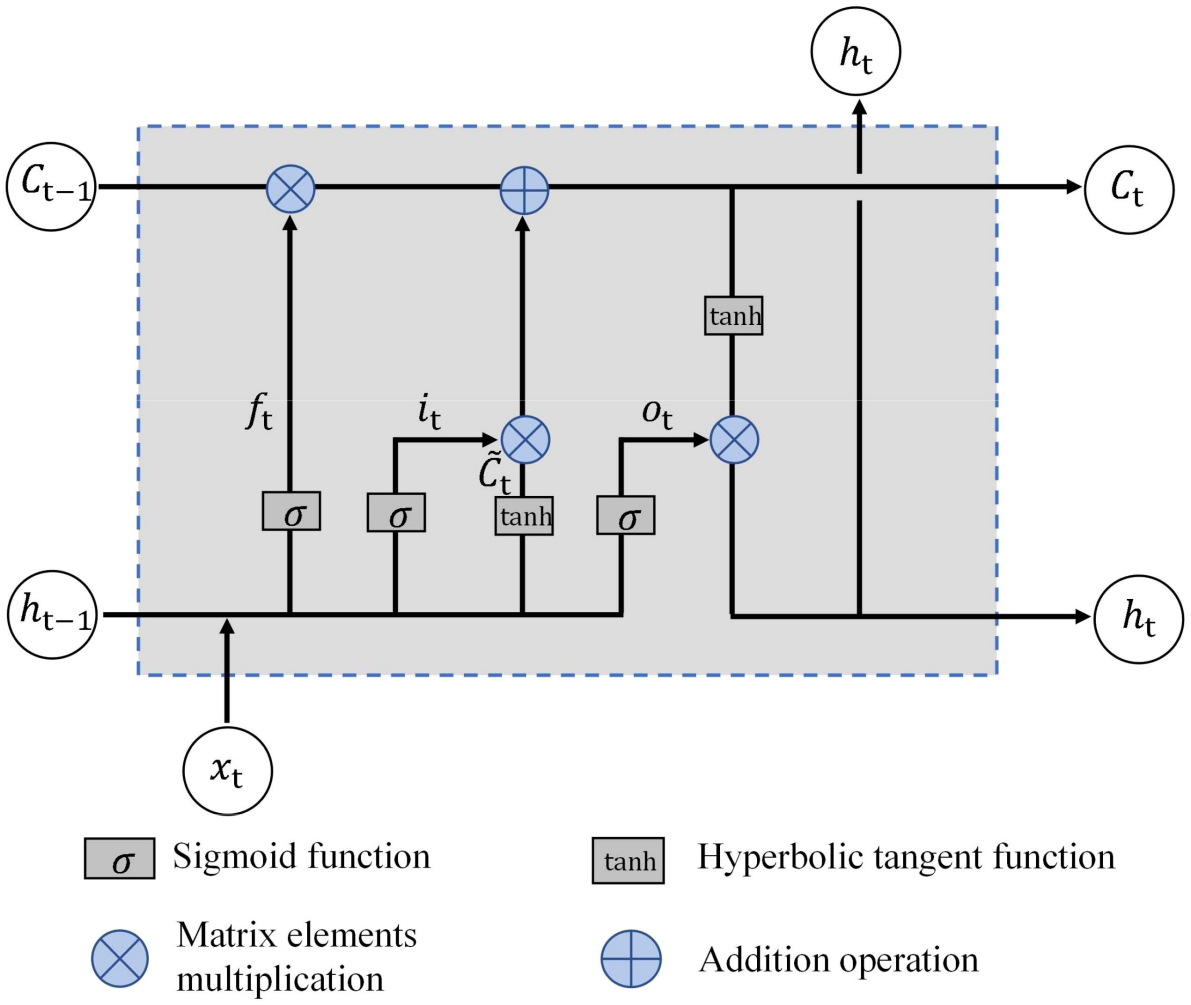
Schematic of the Internal Structure of an LSTM Network.

For a given time step t and input $X_{t}$ , each LSTM unit is defined as follows (**Figure 3**):

(1) Forget gate:


(1) $$f_{t}=\sigma \left(W_{f}\cdot \left[h_{t-1},x_{t}\right]+b_{f}\right)$$

(2) Input gate:


(2) $$i_{t}=\sigma \left(W_{I}\left[h_{t-1},x_{t}\right]+b_{i}\right)$$

(3) Updating unit status:


(3) $${\tilde{C}}_{t}=\tan h\left(W_{C}\left[h_{t-1},x_{t}\right]\right)+b_{C}$$

(4) Unit state:


(4) $$C_{t}=f_{t}*C_{t-1}+i_{t}*{\tilde{C}}_{t}$$

(5) Output gate:


(5) $$o_{t}=\sigma \left(W_{o}\left[h_{t-1},x_{t}\right]+b_{o}\right)$$

(6) Hide status updates:


(6) $$h_{t}=o_{t}*\tan h\left(C_{t}\right)$$

In this context, σ represents the sigmoid function.

BiLSTM computes the bidirectional hidden states by running LSTM layers in both the forward and backward directions along the time axis. The forward LSTM processed the sequence from the first to the last element, whereas the backward LSTM processed it in reverse order. The resulting hidden states from both directions were concatenated to form the final bidirectional hidden state.

#### 2.3.3 Bayesian optimization

Bayesian optimization is an effective global optimization method that is particularly suited for costly black-box objective functions. Hyperparameter optimization for machine learning models guides each iteration by incrementally constructing a probabilistic model, typically a Gaussian process, of the objective function, thereby efficiently identifying the optimal solution.

The key steps involved training an agent model f(x) using existing data points and applying Gaussian process regression to estimate the distribution of the objective function. Subsequently, the next sample point $x_{n+1}$ was selected for evaluation using an Acquisition Function that balances exploration (discovering new regions) and exploitation (leveraging known good regions). The acquisition function we use is Expected Improvement (EI). The Bayesian optimiser estimates the mean and uncertainty of the objective function based on the agent model (Gaussian process) and then selects the location where the Expected Improvement is the largest to be sampled, with the goal of finding the point that maximises the predictive uncertainty of the agent model, usually expressed as:


(7) $$x_{n+1}=arg\underset{x}{\text{ max }}\alpha (x|D_{n})$$

where $\alpha (x|D_{n})$ represents the acquisition function of the current dataset $D_{n}$ . By incorporating new data points in each iteration, Bayesian optimization gradually converges to the global optimal solution. Its advantage is the effective optimization of complex functions with a limited number of evaluations, making it particularly suitable for tasks such as hyperparameter tuning.

The convergence criteria for Bayesian optimization primarily rely on two aspects: optimization iterations and improvement threshold. Optimization iterations involve setting a maximum iteration count, n_iter, at which point the optimization process will terminate once the specified count is reached. In our case, we chose n_iter = 50, indicating 50 optimization iterations. Improvement threshold entails selecting the next sampling point based on the expected improvement criterion from the surrogate model. After a certain number of iterations, if the expected improvement becomes minimal, indicating limited room for further enhancement in the current region, it is considered as convergence, and the optimization process gradually ceases.

**Figure 4:**
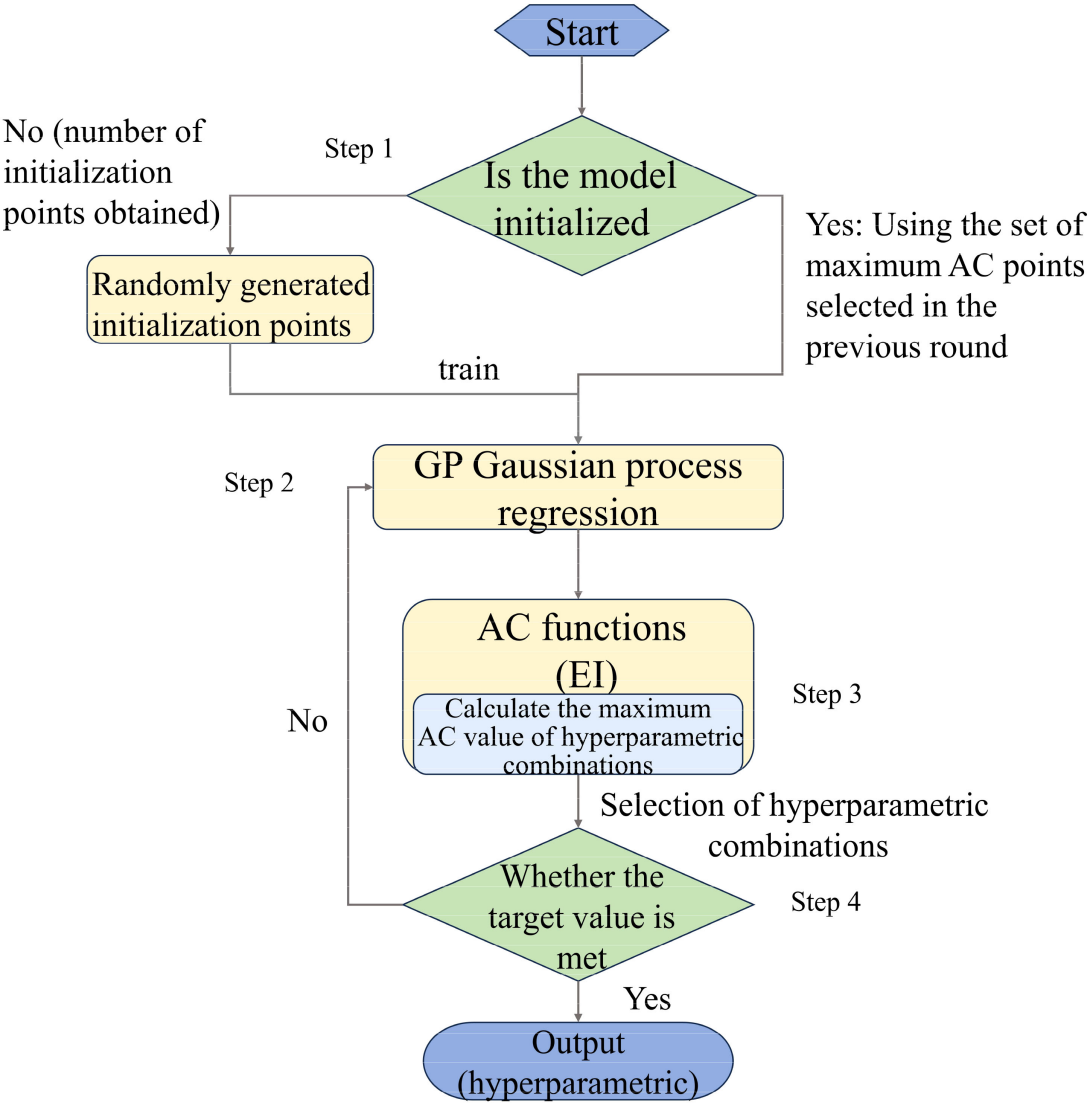
Flowchart of Bayesian optimisation algorithm.

The steps for Bayesian optimization in selecting hyperparameters can be described as follows (**Figure 4**):

Step 1: Initialization: In the initial phase, a certain number of hyperparameter combinations are randomly selected for evaluation (we set init_points = 10, choosing 10 points at random for evaluation).

Step 2: Modeling: A Gaussian process or other surrogate model is used to model the objective function, predicting its shape based on existing evaluation results.

Step 3: Sampling Point Selection: The next sampling point is chosen based on the surrogate model’s predictions and the expected improvement criterion.

Step 4: Evaluation and Update: The selected hyperparameter combination is used to evaluate the objective function (in this context, training and validating the model), and the surrogate model is updated based on the evaluation results.

Step 5: Iteration: Steps 2, 3, and 4 are repeated until the stopping criteria are met (such as maximum iterations or convergence standards).

Compared to traditional grid and random search, Bayesian optimization is more computationally efficient and converges faster. First, it reduces the number of evaluations by using the surrogate model to efficiently explore the parameter space, avoiding redundant calculations in previously evaluated regions. This typically allows it to find optimal hyperparameters with fewer evaluations. Second, it does not rely on exhaustive evaluations, as traditional methods like grid search do, which require evaluations across the entire parameter space. Instead, Bayesian optimization leverages an efficient sampling strategy for global optimization with minimal computational cost. In summary, Bayesian optimization excels at optimizing complex functions within limited evaluations, making it suitable for tasks like hyperparameter tuning. The hyperparameter optimization range for the model in this study can be found in **Appendix S1** in **Supplementary Data Sheet S1**.

#### 2.3.4 Improved BiLSTM network framework for yield estimation

**Figure 5:**
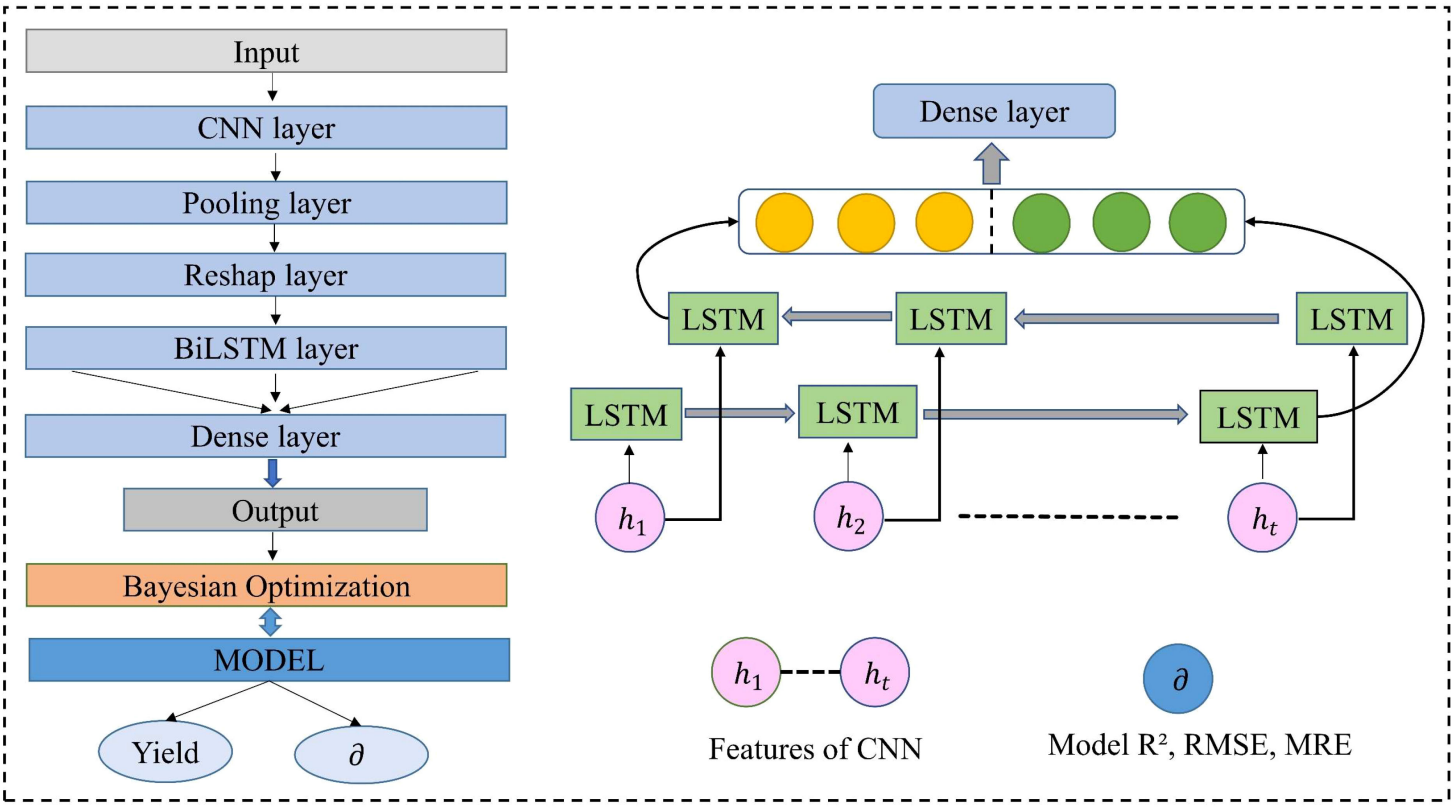
Structure of BCBL model for winter wheat yield estimation and its BiLSTM network structure.

In this study, a BO-CNN-BiLSTM (BCBL) deep learning model was developed for feature extraction from remote sensing, climate, and yield time-series data. The model consisted of an input layer, a convolutional layer (1DCNN), a pooling layer, a reshaping layer, a bidirectional LSTM layer, two fully connected layers, and an output layer and was implemented using the TensorFlow framework, as shown in **Figure 5**. The input features included EVI+LAI (TS), SIF, and climate data, with new features generated through various combinations of these inputs. The target vector was the county-level yield data from 2012 to 2020. Initially, the feature combinations were normalized, and the dataset was randomly split into 80% training and 20% testing sets. Given the significant impact of hyperparameters on model performance, Bayesian global optimization was employed to optimize the hyperparameters, such as the number of units in the convolutional layer, LSTM layer, fully connected layer, and learning rate. Model testing was then conducted using optimal hyperparameter configurations.

#### 2.3.5 Model evaluation and baseline models

Two machine learning models were compared, and deep learning model (LSTM、Transformer) was selected as the baseline to evaluate the accuracy of the models. Random Forest (RF) generates final predictions by constructing multiple independent decision trees and averaging their results, demonstrating strong nonlinear modeling capabilities and being widely used in bagging methods. XGBoost, as a gradient boosting-based algorithm, progressively optimized the model by iteratively constructing decision trees that fit the residuals of previous trees, achieving notable performance in regression and classification tasks. As a baseline, an LSTM model with one LSTM layer and two dense layers was chosen, while a Transformer model with MultiHeadAttention, residual connections, layer normalization, fully connected layers, and dropout was also selected. All models underwent hyperparameter tuning using Bayesian optimization, with the RF and XGBoost results averaged to determine the optimal model after five cross-validations.

To evaluate the estimation performance of the models, three metrics were adopted: coefficient of determination (R²), root mean square error (RMSE), and mean relative error (MRE). The formulas for these metrics are as follows.


(8) $$R^{2}=1-\frac{\sum_{i=1}^{n}{\left(x_{i}-y_{i}\right)}^{2}}{\sum_{i=1}^{n}{\left(x_{i}-\bar{y_{i}}\right)}^{2}}$$

(9) $$RMSE=\;\sqrt{\frac{1}{m}\sum_{i=1}^{n}{\left(x_{i}-y_{i}\right)}^{2}}$$

(10) $$MRE=\;\frac{1}{n}\sum_{i=1}^{n}\frac{x_{i}-y_{i}}{x_{i}}100\%$$

where $x_{i}$ is the statistical yield of wheat; $y_{i}$ is the estimated yield of winter wheat; ${\bar{y}}_{i}$ is the mean statistical yield of wheat; and n is the number of samples in the test set.

## 3 Results and analysis

### 3.1 Model performance using different satellite variables

#### 3.1.1 Effect of single-feature and multi-feature combinations on model performance for crop yield estimation

**Figure 6:**
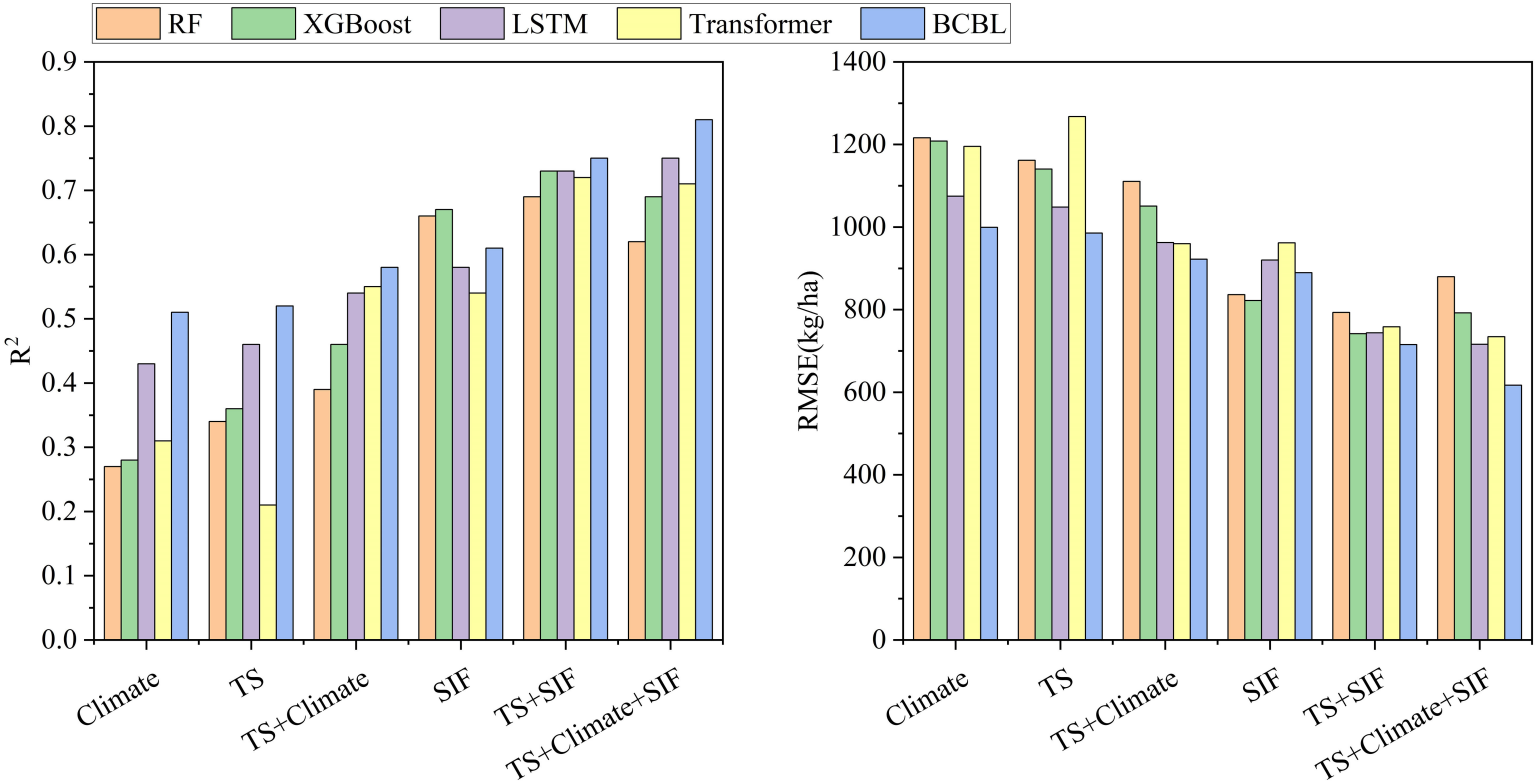
Histograms of yield estimation performance for six models with different combinations of variables, including (Left) R² and (Right) RMSE for four models (RF, XGBoost, LSTM, Transformer and BCBL).

According to the model and dataset combinations outlined in Methods (2.3), SIF outperformed all other models in the single-feature datasets (including TS, climate, and SIF), surpassing both TS and climate. This superior performance was due to the close association of SIF with plant photosynthesis and its rich yield information, which is crucial for accurate crop yield estimation. In contrast, using TS and weather data alone produced similar results but with lower accuracy, likely because of their coarser resolution and mixed image elements. Additionally, comparing the climate data with the TS+climate data revealed only minimal improvements in accuracy, with both the machine learning and deep learning models demonstrating low final accuracies. This indicated that the optimization effect of climate data on TS information was limited, with the contribution of climate data being gradually absorbed by TS data as crop fertility progressed, resulting in minimal improvement. Among all the data combinations, the TS+climate+SIF combination yielded the best performance in the deep learning models, achieving a coefficient of determination (R²) of 0.81 for the BCBL model. Conversely, the TS+SIF combination delivered the best results in the machine learning models, with a coefficient of 0.73 for the XGBoost model (**Figure 6**).

Deep learning can generally excel with large data samples owing to its multilevel architecture, which can learn the deep connections between features, unlike machine learning performing less effectively in this regard. In terms of model accuracy, XGBoost and RF demonstrated superior performance with the TS+SIF dataset. However, adding climate data slightly reduced the accuracy of these models. This decrease may be attributed to two factors: first, the climate data did not significantly enhance the TS dataset in machine learning; second, the vast amount of data and its complex interrelationships limited the ability of machine learning to conduct deep data mining, resulting in poorer performance with the TS+climate+SIF combination.

#### 3.1.2 Enhancement of crop yield estimation model performance by remote sensing indicator SIF

**Figure 7:**
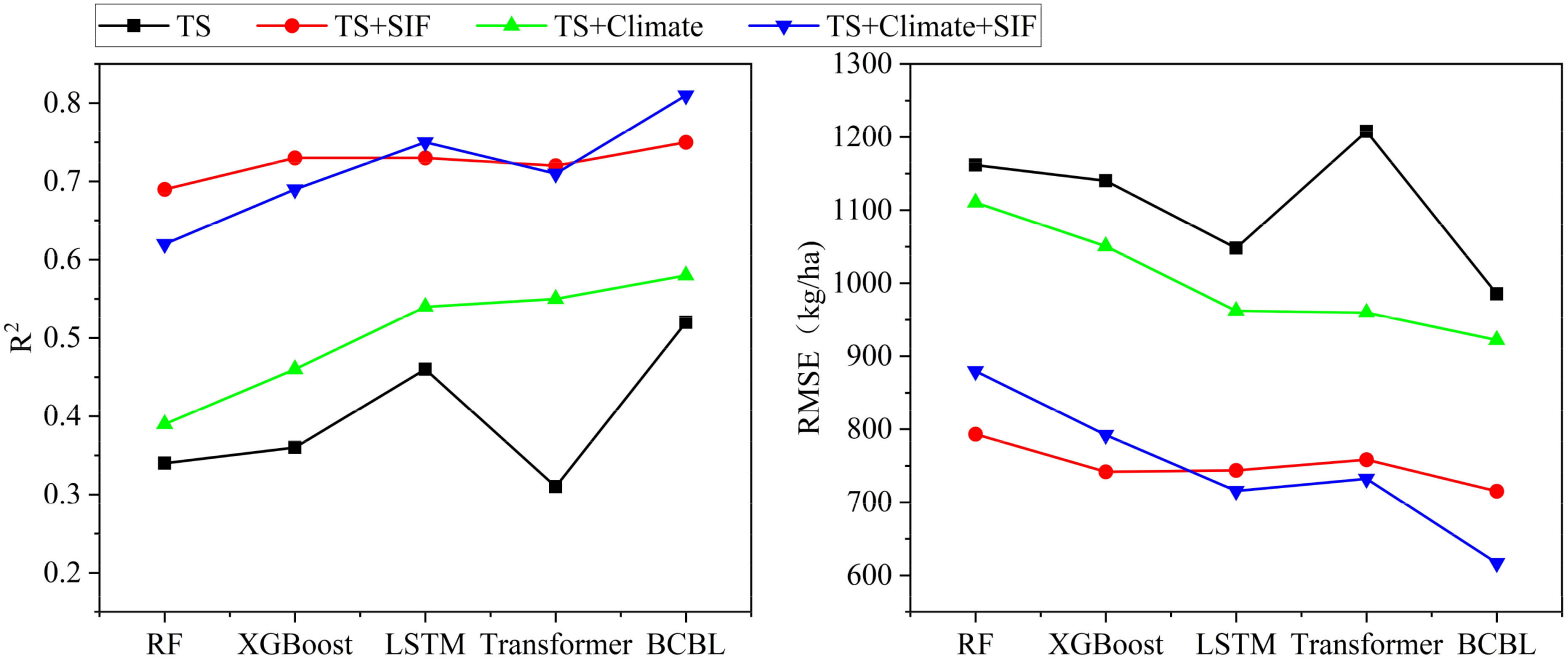
Comparison of the performance improvement (R² and RMSE) of different models using the combination of TS+SIF vs. TS+Climate+SIF data with or without the addition of SIF during the entire study period.

Throughout the study, the accuracy of all four models, whether machine learning or deep learning, was improved to varying extents by incorporating SIF into the TS and TS+climate data combinations (**Figure 7**). Specifically, in the TS+climate+SIF combination, SIF enhanced the R² values of the BCBL, Transformer, LSTM, XGBoost, and RF algorithms by 0.23,0.16, 0.21, 0.23, and 0.23, respectively, while reducing the RMSE by 305.13,227.29, 246.41, 258.40, and 230.70 kg/ha. In the TS+SIF combination, SIF increased the R² values of BCBL, Transformer, LSTM, XGBoost, and RF by 0.23,0.41, 0.27, 0.37, and 0.35, respectively, and reduced the RMSE by 270.11,449.15,304.31, 398.37, and 368.40 kg/ha. These results indicated that SIF significantly complemented the yield estimation as a remote sensing index and enhanced model performance across different datasets. Consequently, BCBL and LSTM exhibiting superior performance were selected for further study.

#### 3.1.3 Comparison of BO- CNN-BiLSTM and LSTM model performance

**Figure 8:**
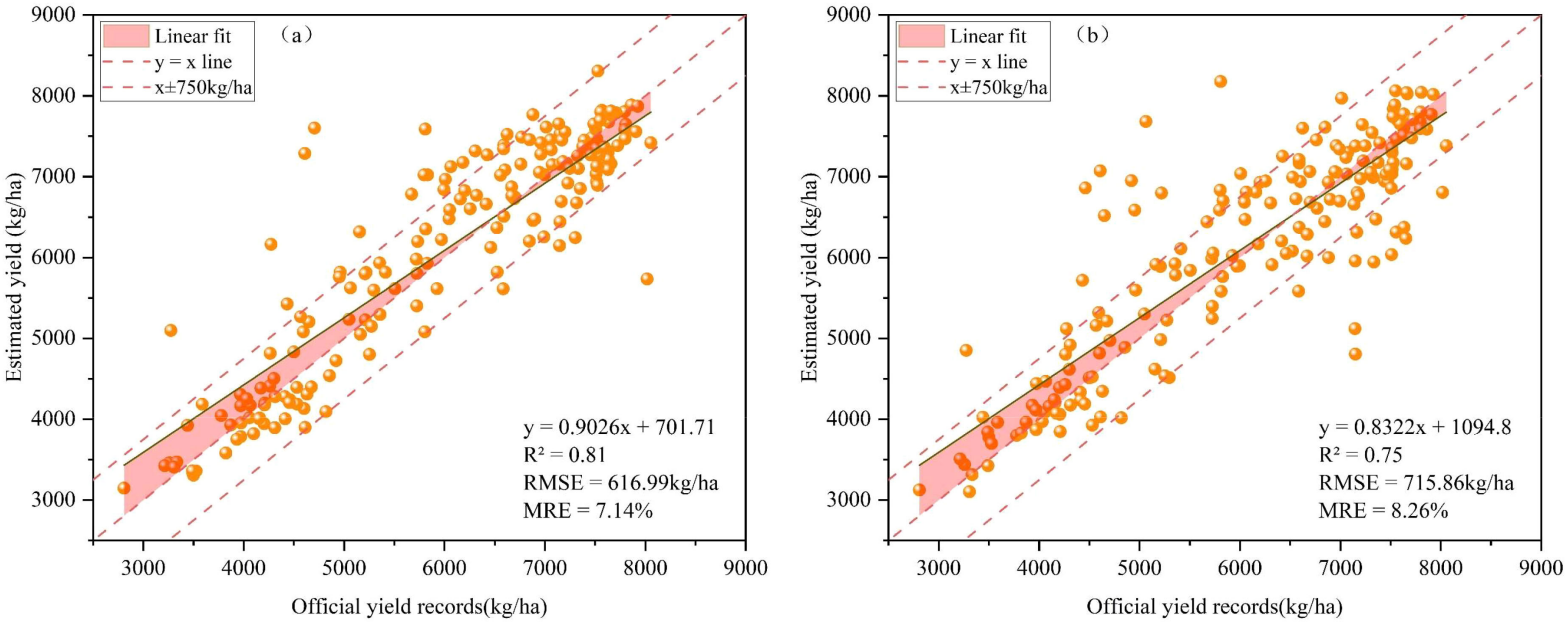
Comparison between BCBL model **(A)** and LSTM model **(B)** in terms of official yield records and test set yield estimation results.

**Figure 8** presents a scatterplot comparing the estimated yields versus the official statistical yields for the BCBL and baseline LSTM models using the same dataset throughout the study period. In the TS+Climate+SIF dataset, the single LSTM model achieved an R² of 0.75, an RMSE of 715.86 kg/ha, and an MRE of 8.26% (**Figure 8B**). In contrast, the BCBL model benefiting from CNN feature extraction and bidirectional LSTM two-way temporal learning achieved an R² of 0.81, an RMSE of 616.99 kg/ha, and an MRE of 7.14% (**Figure 8A**). The BCBL model thus improved R² by 0.06, and reduced RMSE and MRE by 98.87 kg/ha and 1.11%, respectively, compared to the LSTM model. These improvements indicate that the BCBL model significantly enhances yield prediction accuracy and robustness under different data conditions.

The scatterplot shows that the yield estimates of both models are evenly distributed around the 1:1 line, displaying a clear linear relationship, which demonstrates the model’s ability to capture the trend between yield and prediction effectively in most cases. Compared with the single-layer LSTM model, the BCBL model’s scatter points are more concentrated around the 1:1 line, indicating that its predictions are closer to the actual values. Particularly in low- and high-yield areas, the BCBL model significantly reduces overestimation in low-yield regions and underestimation in high-yield regions, resulting in more accurate and stable predictions. By integrating CNN spatial feature extraction with bidirectional LSTM two-way temporal learning, the BCBL model more comprehensively captures the spatiotemporal features of the crop growth process, effectively reducing prediction errors. Further analysis of residuals shows that the absolute values of most residuals are within 750 kg/ha, with only a few counties exceeding this range, indicating the BCBL model’s high accuracy in yield estimation across most areas. However, for counties where residuals exceed 750 kg/ha, data quality issues or model underfitting may be contributing factors. Specifically, high residuals in these counties may result from insufficient inclusion of local meteorological, soil, or other environmental factors in the model, or the model’s weaker generalization ability in specific areas. These outliers indicate that although the overall performance of the BCBL model is better than that of LSTM, there is still room for further optimization in the future to better handle the regional variability problem.

In summary, the BCBL model demonstrates a clear advantage over the LSTM model in yield prediction, with smaller errors, and most of the estimated value points are near the 1:1 line, thus reflecting the trend of yield more reliably, and having an improved effect on the underestimation of high yield and overestimation of low yield.

### 3.2 Early stage estimation capability of the model

**Figure 9:**
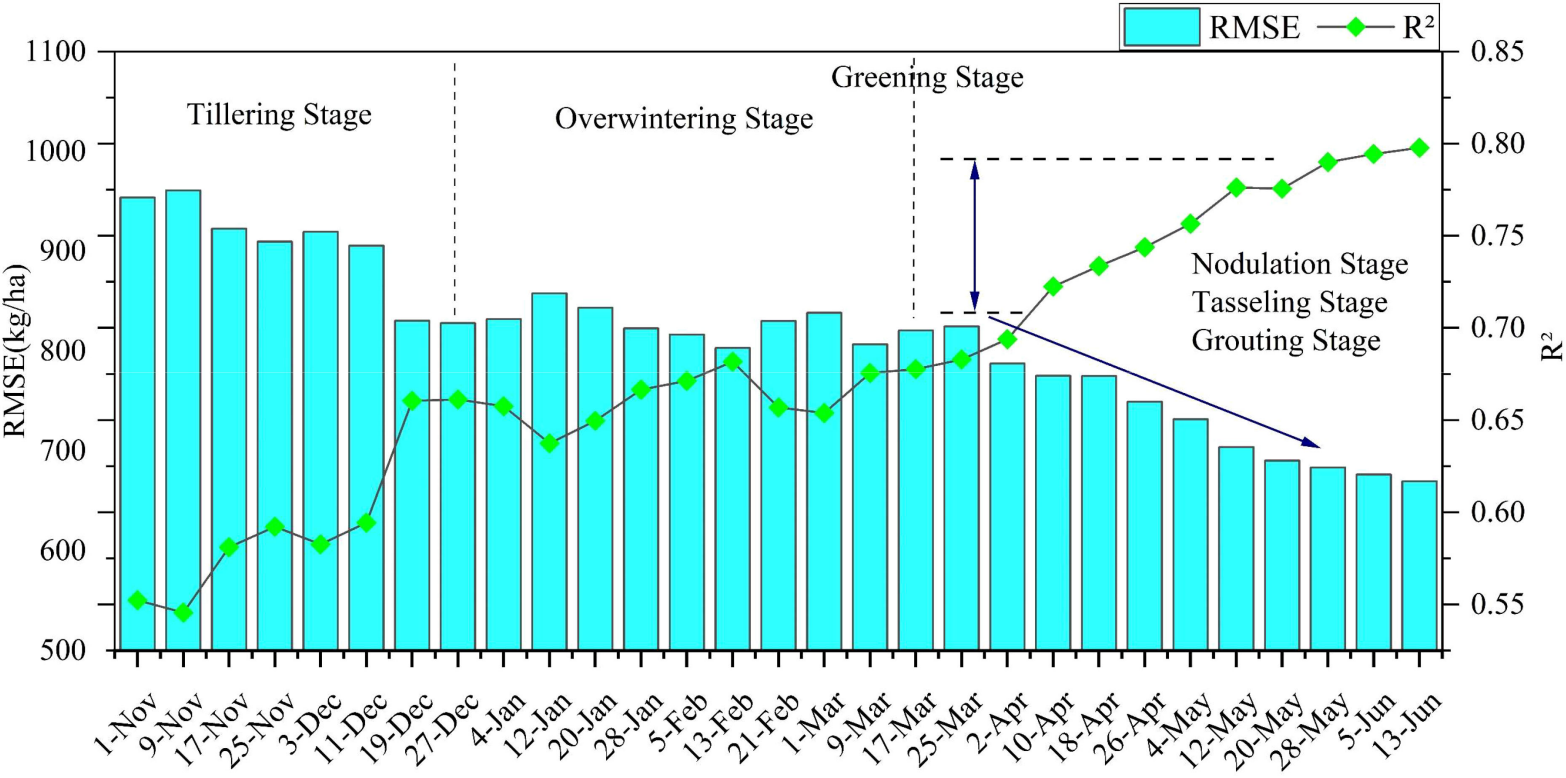
Analysis of the change in accuracy of the BCBL model at different growth stages evaluated based on R² and RMSE metrics.

By progressively inputting the features to analyze the timeliness and accuracy of yield estimation, it is possible to evaluate the model’s early stage yield prediction capabilities and assess the significance of the data for each period in the winter wheat growth cycle. The BCBL model exhibiting the highest accuracy was used to analyze its performance from 2010 to 2020 using the TS+Climate+SIF data combination. Overall, the estimation error of the model decreased and stabilized as more data input was incorporated. **Figure 9** illustrates the improved estimation performance of the BCBL model with data accumulation. The model initially performed poorly until January owing to the slow growth of winter wheat and insufficient time-series inputs during the early seeding and emergence stages, leading to lower estimation accuracy during this period. From winter to mid-March of the following year, the model’s accuracy improved, with R² stabilizing at approximately 0.65. This stabilization was attributed to the nearly halted growth of winter wheat during the overwintering period, which weakened the correlation between remote sensing variables and yield. Despite the stability of the model, its accuracy remained insufficient for a precise yield estimation. However, from mid-March to mid-May, as temperatures rose and winter wheat progressed to the nodulation and spiking stages, model performance significantly improved with the accumulation of variables. During this period, R² increased from 0.67 to 0.77, and RMSE decreased from 820.44 to 703.79 kg/ha. The most significant changes in R² and RMSE during this stage indicate that the information provided by remote sensing variables is crucial for yield estimation. This period, characterized by rapid wheat growth, increased chlorophyll content, enhanced photosynthesis, and high nutrient and water demands, is critical for yield accumulation.

Overall, the model achieved an R² of 0.77 and an RMSE of 703.79 kg/ha in early May, providing accurate yield estimates approximately 25 d before the winter wheat harvest.

### 3.3 Spatial and temporal distribution of wheat yield estimation

**Figure 10:**
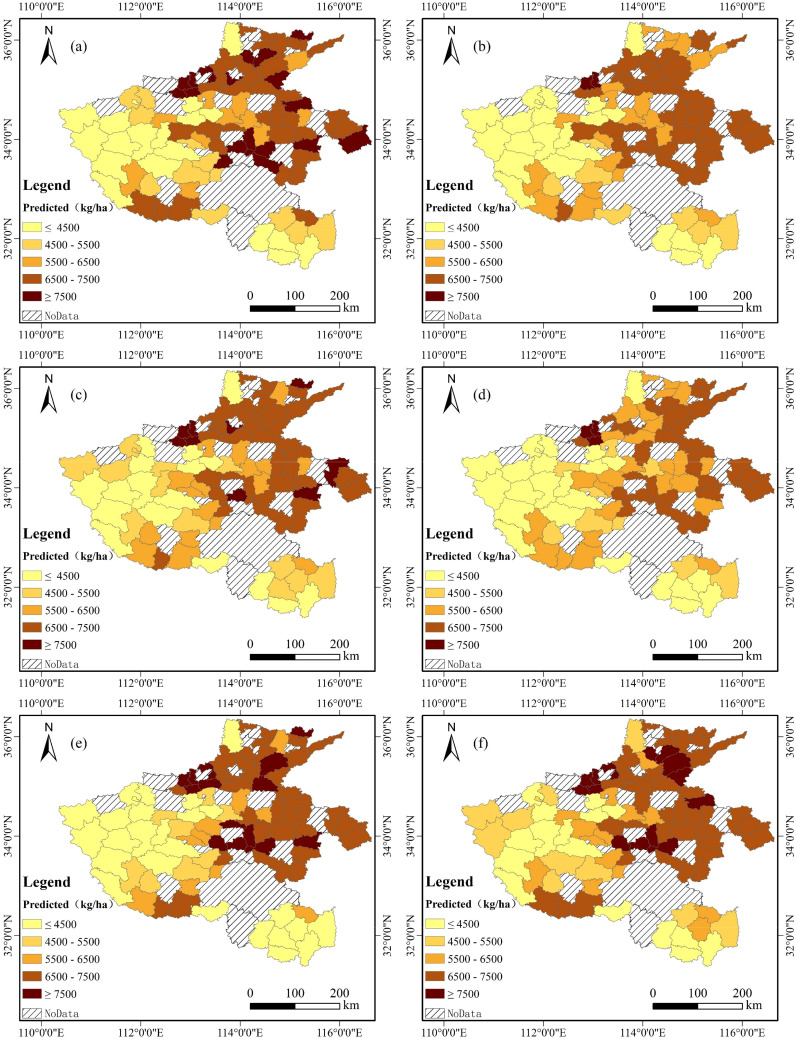
Temporal and spatial patterns of winter wheat production estimates for 2015–2020: **(A)** 2015, **(B)** 2016, **(C)** 2017, **(D)** 2018, **(E)** 2019, and **(F)** 2020.

The spatial distribution of end-of-season wheat production in Henan Province from 2015 to 2020, estimated using the TS + Climate + SIF data combination and BCBL model (**Figure 10**), aligned closely with the official statistics. Wheat yields were generally lower in the western region and higher in the eastern and northern regions, reflecting the impact of topographic relief. The higher elevations in the west and the central and northern plains were more suitable for wheat cultivation, whereas the limited cultivation in the southern Xinyang region resulted in lower yields owing to topographical and climatic factors. The yield fluctuations were more pronounced in medium- and high-yielding regions, whereas the overall trend demonstrated stability and growth. In 2018, the yields declined in some areas due to late frost and downy mildew, whereas favorable climatic conditions in 2019–2020 led to abundant wheat harvests. Overall, the spatial and temporal distribution of winter wheat production in Henan Province from 2015 to 2020 based on the BCBL model was consistent with the official statistical results.

### 3.4 Predictive capability of the BCBL model under drought conditions

**Figure 11:**
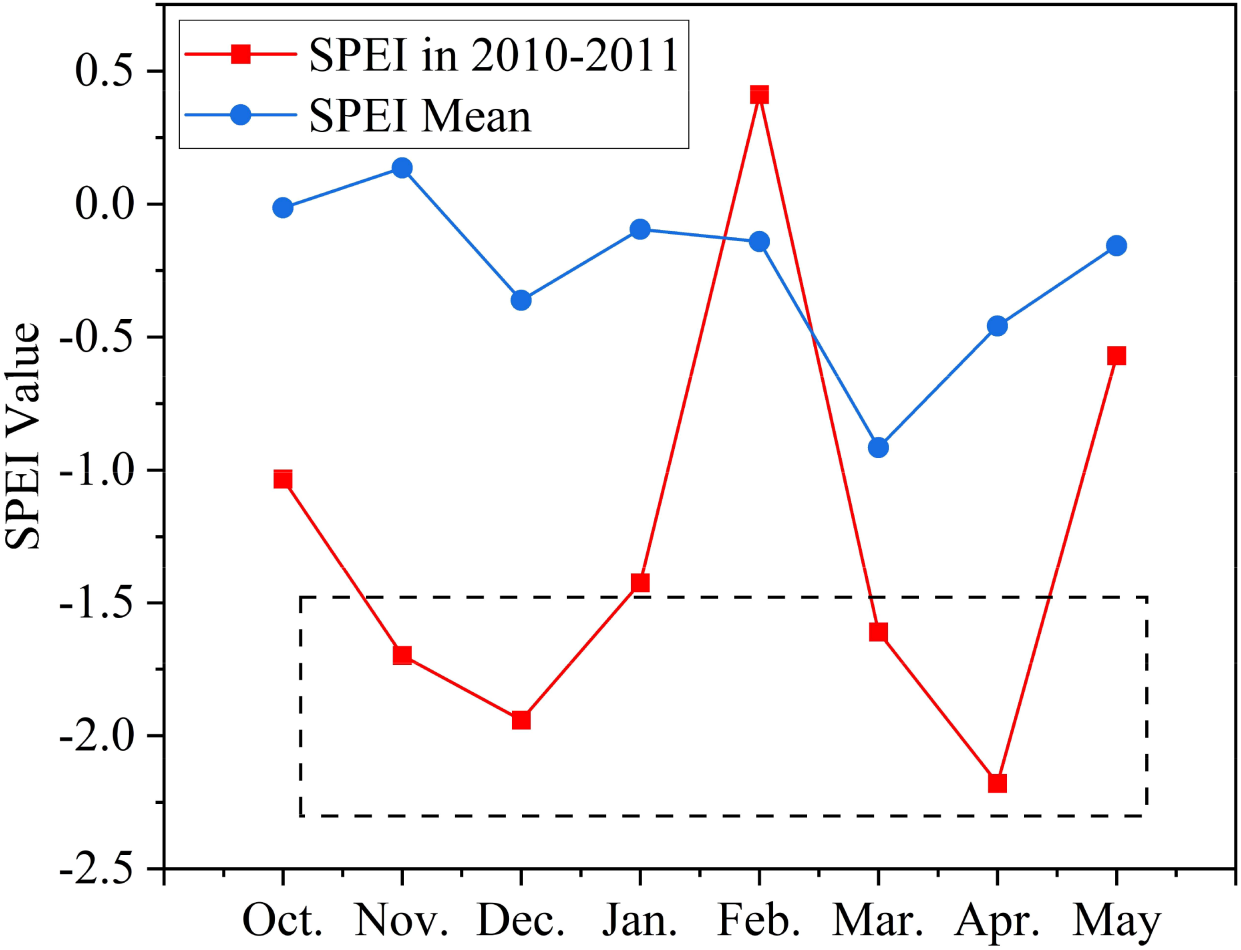
SPEI Trend from October 2010 to May 2011(SPEI in 2010–2011: Mean value for the study area in that year, SPEI Mean: 10-year mean value for the study area).

Based on the adopted classification criteria for the SPEI dataset(**Table 1**), we compared the monthly mean SPEI values for Henan Province over the study period, considering past drought conditions in the region. The results (**Figure 11**) indicate that during the 2010–2011 winter wheat growing season, Henan Province experienced moderate drought (October to January) followed by severe drought (March to May). Using the drought conditions of 2010–2011 as a case study, we evaluated the robustness of the model’s yield predictions under drought stress.

Using year-by-year cross-validation, we selected 2011 as the test set and 2012-2020 as the training set. The results showed that the model performed poorly in 2011 under drought conditions when only TS and meteorological data were used, with an R² of 0.46 and RMSE of 1115.27 kg/ha, which was lower than the model performance across the entire study period (R² range: 0.55-0.79; RMSE range: 653.18-960.82 kg/ha). This indicates that the model’s overall predictive performance deteriorates under drought conditions. However, when SIF was included as supplementary data, the model’s overall performance improved significantly. The combination of TS, meteorological data, and SIF in the BCBL model led to an increase in R² by 0.19 and a reduction in RMSE by 210.76 kg/ha. Although the model’s precision remained lower than the average accuracy for the entire study period, the inclusion of SIF notably enhanced performance.

These results highlight the unique advantage of SIF in drought monitoring, especially when integrated with the BCBL model. SIF directly reflects changes in plant photosynthesis, capturing the physiological state of plants under water stress, with a signal closely associated with plant growth. This coupling is particularly crucial in drought environments, as drought inhibits plant photosynthesis and affects SIF values. By integrating SIF with TS and meteorological data, the model’s robustness in drought conditions was strengthened, allowing for a more accurate capture of plant physiological responses and improved yield prediction accuracy. This finding not only underscores the importance of SIF in crop yield prediction but also emphasizes its potential application in drought detection, further validating the suitability of the SIF-based BCBL model under complex climate conditions.

## 4 Discussion

### 4.1 Impact of BCBL model on wheat yield estimation

In addition to leveraging multisource data fusion to enhance the estimation accuracy, advancements in computer science have introduced new methods for estimating crop parameters. Deep-learning techniques can automatically extract useful features from raw data and construct complex models that capture intricate patterns. In this study, the BCBL, Transformer and LSTM deep learning models demonstrated superior performance compared with traditional machine learning models, such as RF and XGBoost. Previous studies have demonstrated that LSTM models can achieve higher accuracy in winter wheat yield estimation than RF and XGBoost models (Cao et al., 2021, 2022; Gong et al., 2023). As a variant of the recurrent neural network (RNN), LSTM excels with time-series data. In contrast, the Transformer model processes global dependencies between each element in the sequence using its self-attention mechanism, often demonstrating significant advantages in many traditional time series tasks. Meanwhile, machine learning models often struggle due to their inability to effectively recognize complex nonlinear relationships in large datasets, leading to reduced accuracy. In this study, the multilevel mixed model (BCBL) demonstrated the superior estimation and high generalizability, achieving the optimal performance with an R² of 0.81, RMSE of 616.99 kg/ha, and MRE of 7.14% for different growth stages of winter wheat. The BCBL model can benefit from the information processing advantages of CNN and BiLSTM. The 1DCNN can efficiently extract local features from the sequence data, whereas BiLSTM captures bidirectional contextual information, allowing for richer feature representations. Unlike unidirectional LSTM, BiLSTM considers both forward and backward sequence information, thereby enhancing its ability to capture long-term dependencies with greater accuracy. In addition, Bayesian optimization can identify the best hyperparameters for varying sample sizes, thereby improving the model’s applicability and resilience to interference. Consequently, in this study, the BCBL model outperformed other models (LSTM, RF, and XGBoost) in terms of yield estimation accuracy across different data combinations and performed well even under abnormal environmental conditions.

Additionally, to analyze the model’s performance across different regions and climate conditions and demonstrate its robustness and applicability, we conducted cross-regional validation experiments. Specifically, we applied the model to data from various geographic regions and climate zones to further verify its generalizability and adaptability, ensuring its suitability in different environments. The details are as follows: following the dataset preparation and preprocessing steps in Section 2.2, we selected three counties in Shandong Province from south to north—Juye County, Pingyin County, and Guangrao County—as test areas for model generalization. Using the BCBL model, we estimated yields from 2011 to 2020 based on relevant data, keeping the model structure and parameters unchanged. The predictive accuracy of the model over this ten-year period is shown in **Table 2**.

**Table 2:** Estimated yields of winter wheat, 2011-2020 (Juye County, Pingyin County, Guangrao County).

Year	Juye Country			Pingyin Country			Guangrao County		
	OY	EY	AC	OY	EY	AC	OY	EY	AC
2011	5982.00	5515.33	92.20%	5289.32	4858.18	93.74%	–	–	–
2012	5595.50	5581.36	93.09%	5379.58	5233.53	97.29%	7022.00	6043.81	86.07%
2013	6220.55	5806.00	93.34%	5065.79	5129.78	101.26%	6862.00	5880.17	85.69%
2014	6298.56	5256.34	83.45%	5076.48	5176.06	101.96%	6908.00	5895.60	85.34%
2015	6519.32	5518.70	84.65%	5231.59	5308.63	101.47%	6702.00	5667.40	84.56%
2016	6594.21	5468.75	82.93%	5186.58	5358.89	103.32%	6325.00	5497.64	86.92%
2017	6672.25	5633.01	84.42%	5075.33	5403.32	87.74%	6363.00	5434.58	85.41%
2018	5842.56	5144.94	88.06%	4825.31	5247.12	108.74%	5965.00	5322.84	89.23%
2019	6183.53	5420.34	87.66%	–	–	–	5292.00	5533.20	104.56
2020	6382.81	5455.10	85.31%	–	–	–	6506.00	5396.51	82.95%

OY, Official Yield(kg/ha); EY, Estimated Yield (kg/ha); AC, Accuracy (%); “-”: Nodata.

Based on the estimation results, we found that the accuracy of the model’s extrapolation declined to varying degrees, which is consistent with most empirical models. Yield prediction results for the period from 2011 to 2020 indicate that the model has good adaptability across different regions and years, achieving an average accuracy of 90.95%, with a maximum annual prediction error of 17.07%. However, there were instances of yield overestimation or underestimation in certain years, which may reflect significant differences in the feature distributions of training data from Henan and Shandong. Specifically, climate conditions, soil types, and crop management practices vary widely between these two regions, potentially causing the model to favor the feature patterns of one region when learning feature relationships. When the model performs well on training data from one region, applying it to another region with differing feature distributions can result in inconsistent prediction outcomes, evidenced by decreased accuracy when applied outside the region it was trained on. This phenomenon suggests that differences in feature distributions can pose a major challenge to prediction accuracy when applying the model across regions and highlights the need to consider data distribution consistency in multi-regional or cross-crop applications. Overall, the BCBL model can adapt to inter-annual fluctuations in climate change and agricultural management practices, maintaining high predictive accuracy under varying data conditions and providing foundational support for future applications in other regions or different crops.

### 4.2 Advantages of multi-source remote sensing data fusion in yield estimation

The results indicated that the multisource remote sensing data fusion (TS + Climate) consistently provided better yield estimation throughout the wheat growth stages than the single remote sensing data (TS or Climate) alone (**Figure 6**). The model effectively identified yield accumulation under varying growth conditions, with different types of remote sensing data reflecting various aspects related to yield. During the early growth stages, soil moisture, nutrient indices, and climate data, such as rainfall and temperature, are critical for assessing pre-emergence growth. During the middle and late growth stages, parameters such as EVI and LAI derived from different spectral bands are crucial for evaluating wheat growth and development. In the late growth stage, as the leaf development becomes more abundant, the temperature and water stress may affect the growth, with the climate data providing essential contextual support to explain the changes in crop health due to weather anomalies. As climate data, such as temperature, precipitation, and wind speed, were progressively integrated with remotely sensed data, the model could leverage the synergistic effects between these data sources to capture the real-time state of the crop, particularly in areas with lush vegetation, where spectral saturation could lead to issues with saturated EVI. Furthermore, previous studies have demonstrated that SIF is closely related to crop photosynthesis (Xu et al., 2023) and is more sensitive to drought conditions, thus offering unique and complementary remote sensing data for assessing photosynthetic information. In this study, we quantified the effect of SIF on the yield estimation across various data combinations, indicating that SIF increased the R² by 0.21 to 0.37 and decreased RMSE by 246.41 to 398.37 kg/ha in different models, thereby improving the estimation accuracy. The estimated yields were generally consistent with official statistics. This enhancement was attributed to the ability of SIF to accurately reflect plant photosynthetic activities. When combined with other remote sensing data (TS, Climate) in multisource data fusion, SIF provides more comprehensive information on crop growth and yield prediction. This data fusion enhanced the capacity of the model to identify the factors influencing yield formation during critical, climate-sensitive periods, thereby improving the overall accuracy of yield estimates.

### 4.3 Computational cost and speed of inference for BCBL models

In practice, the extensive application of the BCBL deep learning model is also influenced by computational costs and inference speed, especially in large-scale applications. This model combines Convolutional Neural Networks (CNN) and Bidirectional Long Short-Term Memory networks (BiLSTM), which, despite its excellent predictive accuracy, presents challenges in terms of computational cost and inference speed. In the BCBL, Bayesian Optimization (BO) is used during training to tune hyperparameters, which can improve model performance; however, the optimization process itself is computationally intensive, particularly with large datasets. Each optimization cycle requires multiple model training iterations, significantly increasing training time and computational load. Furthermore, as model complexity (such as the addition of more CNN and BiLSTM layers) increases, so does the computational cost. During inference, while the BCBL model has an advantage in accuracy, its inference speed is affected by model structure and computational resources. The computational complexity of the bidirectional LSTM layer is high and the inference process may take longer. In addition, the real-time application of the model faces hardware resource limitations, especially in scenarios requiring real-time prediction, where the inference speed may become a bottleneck.

Nevertheless, with advances in computational power and hardware acceleration technologies, the issues of computational cost and inference speed in the BCBL are expected to improve. For example, utilizing GPU or TPU acceleration for training, as well as applying techniques like quantization and pruning to optimize inference efficiency, can significantly reduce computational resource demands and improve inference speed. These developments may lower the application barrier for this technology, promoting wider adoption of the BCBL in large-scale applications.

### 4.4 Future research

The results of this study demonstrated that the multisource remote sensing fusion method effectively integrated complementary information from various data types, thereby enhancing yield estimation accuracy (**Figures 6**, **7**). This study primarily examined the cumulative effect of county-level yields using remotely sensed time-series data. Previous research has indicated that incorporating soil properties and spatial information, such as elevation and latitude, can potentially improve the model accuracy (Wang et al., 2020, 2023; Tian et al., 2021). Future research should explore how the spatial and temporal heterogeneity of remotely sensed data, including factors such as topography and the location of neighboring counties, affects yield data. Additionally, enriching feature dimensions could help explain variations in yield across different ecosystems and further improve yield estimation accuracy. A deeper understanding of the mechanisms underlying SIF can enhance the information available for crop yield estimation. Liu et al. (2022) demonstrated that the machine learning models incorporating SIF data achieved the highest R² values than other remotely sensed variables like NDVI, EVI, and NIR_v_ (Liu et al., 2022). The SIF data used in this study were sourced from the global daylight-induced chlorophyll fluorescence dataset (0.05°, 1d) provided by Yao (2021). Future research can explore the application of different SIF products, such as SIF-GPP, SIF-NPP, and SIF_yields_, which are gaining attention for their potential to improve crop productivity and yield estimates based on crop light-use efficiency. Current satellite fluorescence detection platforms include GOSAT (TANSO FTS), Sentinel-5P (TROPOMI), US OCO-2, Japan’s GOSAT-2, and China’s TanSat. These platforms have generated extensive fluorescence data, advancing research and applications related to SIF. However, the coarse resolution of the SIF data products used in this study limited field-scale research. In the future, higher-resolution products (e.g., 300m FLEX) can be more appropriate for regional crop growth monitoring and yield prediction at the farm level, offering stronger support for precision agriculture.

## 5 Conclusion

In this study, a BO-CNN-BiLSTM (BCBL) model was developed to estimate the winter wheat yield in Henan Province by combining remote sensing data (EVI, LAI, and SIF) with climate data. The BCBL model outperformed the traditional RF, XGBoost, and single LSTM models, achieving the highest yield estimation accuracy with an R² of 0.81 and an RMSE of 616.99 kg/ha when using the combination of TS+SIF+climate data. The BCBL model demonstrated a strong generalization ability and stability in the spatiotemporal distribution of yield estimation across different years, with SIF data performing exceptionally well as a supplement to traditional remote sensing data in various feature combinations. The model successfully identified the critical fertility period of winter wheat from mid-March to mid-May, achieving stable yield estimation approximately 25 d before harvest. This deep learning model based on multisource data fusion exhibited excellent spatiotemporal accuracy in county-level yield estimation. In the future, the BCBL model can be extended and validated in other regions and for different crop types to explore its applicability and limitations. Additionally, more remote sensing data sources, such as soil moisture and surface temperature, can be incorporated to enrich input features. To achieve true real-time application, this model should be integrated with real-time remote sensing monitoring systems, enabling dynamic monitoring of crop growth processes and early warning capabilities, thereby providing more timely support for agricultural production and decision-making.

## Data Availability

The original contributions presented in the study are included in the article/**Supplementary material**. Further inquiries can be directed to the corresponding author.
