# Supplementary material for: BO-CNN-BiLSTM deep learning model integrating multisource remote sensing data for improving winter wheat yield estimation
Source: Front Plant Sci. 2024 Dec 20;15:1500499. doi: 10.3389/fpls.2024.1500499 (PMC11695193; doi:10.3389/fpls.2024.1500499)
Supplement: Supplementary file 1 [file DataSheet1.pdf]

Appendix: Model Hyperparameter Optimization and Ranges

In this study, we performed hyperparameter optimization for several models. Below is a detailed description of each model and its corresponding hyperparameter optimization ranges.

1. CNN-BiLSTM Model Hyperparameter Ranges

The CNN-BiLSTM model combines the strengths of Convolutional Neural Networks (CNN) and Bidirectional Long Short-Term Memory (BiLSTM) networks, making it well-suited for handling sequential data. The hyperparameter optimization ranges are as follows:

filters: Number of filters in the convolutional layer, range (32, 128)

kernel_size: Size of the convolution kernel, range (2, 5)

pool_size: Pooling layer size, range (2, 5)

lstm_units: Number of units in the LSTM layer, range (64, 256)

dense_units_1: Number of units in the first dense layer, range (16, 64)

dense_units_2: Number of units in the second dense layer, range (8, 32)

learning_rate: Learning rate, range (0.0001, 0.01)

2. LSTM Model Hyperparameter Ranges

The LSTM model is used for time-series data processing. Its hyperparameter optimization ranges are as follows:

lstm_units: Number of units in the LSTM layer, range (32, 256)

dense_units_1: Number of units in the first dense layer, range (16, 64)

dense_units_2: Number of units in the second dense layer, range (8, 32)

learning_rate: Learning rate, range (0.0001, 0.01)

3. Transformer Model Hyperparameter Ranges

The Transformer model is a powerful sequence-to-sequence architecture widely used in natural language processing (NLP) tasks and other sequential data tasks. Its hyperparameter optimization ranges are as follows:

num_heads: Number of heads in the self-attention mechanism, range (1, 8)

num_layers: Number of encoder (or decoder) layers in the Transformer, range (1, 4)

hidden_space: Dimension of hidden units in each layer, range (32, 256)

dropout_rate: Dropout rate during training to prevent overfitting, range (0.1, 0.5)

learning_rate: Learning rate, range (0.0001, 0.01)

4. XGBoost Model Hyperparameter Ranges

XGBoost is a machine learning algorithm based on gradient-boosted trees, commonly applied to classification and regression tasks. Its hyperparameter optimization ranges are as follows:

max_depth: Maximum depth of the trees, range (2, 10)

learning_rate: Learning rate, range (0.001, 0.1)

max_leaves: Maximum number of leaves per tree, range (2, 150)

n_estimators: Number of weak classifiers, range (10, 1000)

5. Random Forest (RF) Model Hyperparameter Ranges

Random Forest is an ensemble learning method that makes predictions by constructing multiple decision trees. Its hyperparameter optimization ranges are as follows:

n_estimators: Number of decision trees, range (10, 1000)

max_depth: Maximum depth of the trees, range (3, 20)

max_features: Maximum number of features considered for splitting, range (1, 10)

min_samples_split: Minimum number of samples required to split an internal node, range (1, 10)

min_samples_leaf: Minimum number of samples required at a leaf node, range (1, 10)
